# Renewal of Prescriptions by Druggists

**Published:** 1868-06

**Authors:** 


### Renewal of Prescriptions by Druggists.

Various societies in America have now passed resolutions respecting the practice of druggists renewing prescriptions without authority; and the Medical Societies of the City and County of New York have notified the same to the druggists of the city; and the following legal opinion has been given on the subject by Mr. J. D. Harnett, attorney and counsellor-at-law:

"In answer to your inquiry, 'Have physicians a right of property in the prescription given by them to their patients?' I state, first, the prescription is a direction from a physician to some druggist to put up for and prepare for the patient's use, a certain medicine. When the druggist performs this act, and files away the prescription, he has no right to again put up and prepare medicine from that prescription, unless he do so by the orders of the physician who originally gave it. He has no more right to do so, than a merchant would have to deliver, on a written order for one barrel of flour, sundry barrels after the one called for had been delivered. A more important feature is, however, involved in the matter of physicians' prescriptions being duplicated by a druggist without the physician's authority or instruction, which is, that the medicine so duplicated may be entirely unsuited to the patient's changed condition of health, of which the druggist has no opportunity of knowing. No one is capable of judging in such matters but the attending physician. The druggist who duplicates a physician's prescription without the physician's orders, commits a crime against society, inasmuch as he permits medicine to leave his store which may cause the death of the person to whom it is administered. Second: medical societies have a right, (and, indeed, I think it is a duty which they should attend to) to prescribe and establish a rule for the government of druggists in such matters, which, no doubt, druggists would carefully observe. This would save the medical profession from many charges of mal-practice, and many persons from the injuries resulting from the continued use of a medicine not advised or prescribed by a physician."—*British Medical Journal.*

